# Predicting drug-free remission in rheumatoid arthritis: A prospective interventional cohort study

**DOI:** 10.1016/j.jaut.2019.06.009

**Published:** 2019-12

**Authors:** Kenneth F. Baker, Andrew J. Skelton, Dennis W. Lendrem, Adam Scadeng, Ben Thompson, Arthur G. Pratt, John D. Isaacs

**Affiliations:** a Musculoskeletal Research Group, Institute of Cellular Medicine, Newcastle University, Newcastle upon Tyne, United Kingdom; b Musculoskeletal Unit, Newcastle upon Tyne Hospitals NHS Foundation Trust, Newcastle upon Tyne, United Kingdom; c Bioinformatics Support Unit, Newcastle University, Newcastle upon Tyne, United Kingdom

**Keywords:** Rheumatoid arthritis, Drug-free remission, Disease-modifying anti-rheumatic drug, Biomarker, Cessation, CD4^+^ T cell

## Abstract

**Background:**

Many patients with rheumatoid arthritis (RA) achieve disease remission with modern treatment strategies. However, having achieved this state, there are no tests that predict when withdrawal of therapy will result in drug-free remission rather than flare. We aimed to identify predictors of drug-free remission in RA.

**Methods:**

The Biomarkers of Remission in Rheumatoid Arthritis (BioRRA) Study was a unique, prospective, interventional cohort study of complete and abrupt cessation of conventional synthetic disease-modifying anti-rheumatic drugs (DMARDs). Patients with RA of at least 12 months duration and in clinical and ultrasound remission discontinued DMARDs and were monitored for six months. The primary outcome was time-to-flare, defined as disease activity score in 28 joints with C-reactive protein (DAS28-CRP) ≥ 2.4. Baseline clinical and ultrasound measures, circulating inflammatory biomarkers, and peripheral CD4^+^ T cell gene expression were assessed for their ability to predict time-to-flare and flare/remission status by Cox regression and receiver-operating characteristic (ROC) analysis respectively.

**Results:**

23/44 (52%) eligible patients experienced an arthritis flare after a median (IQR) of 48 (31.5–86.5) days following DMARD cessation. A composite score incorporating five baseline variables (three transcripts [*FAM102B*, *ENSG00000228010*, *ENSG00000227070*], one cytokine [interleukin-27], one clinical [Boolean remission]) differentiated future flare from drug-free remission with an area under the ROC curve of 0.96 (95% CI 0.91–1.00), sensitivity 0.91 (0.78–1.00) and specificity 0.95 (0.84–1.00).

**Conclusion:**

We provide proof-of-concept evidence for predictors of drug-free remission in RA. If validated, these biomarkers could help to personalize immunosuppressant withdrawal: a therapy paradigm shift with ensuing patient and economic benefits.

## Highlights

• Treatment may be safely stopped in 50% of rheumatoid arthritis remission cases.
• Drug-free remission is predicted by clinical, serum and transcriptional biomarkers.
• Validation of our findings will guide personalised treatment withdrawal in RA.

## 1 Introduction

The past two decades have witnessed a remarkable revolution in rheumatoid arthritis (RA) outcomes, from a disease of inexorable joint destruction and disability to one where sustained remission is now a realistic and achievable treatment target [1]. Many of these advances have been realised through the effective use of disease-modifying anti-rheumatic drugs (DMARDs), especially their initiation in the early phases of disease and escalation in a treat-to-target fashion [2].

Although transformative for patients living with RA, the use of DMARDs comes at a price. Severe life-threatening toxicity is possible, including bone marrow suppression and hepatotoxicity [3]. Less severe but equally debilitating adverse effects are frequently encountered, such as nausea. Furthermore, the prescription and safety monitoring requirements are costly for healthcare providers and intrusive to patients’ lifestyles. There are thus several motivations to consider DMARD minimisation in the setting of RA remission, a concept which is now recognised in international RA management guidelines [1,4]. Indeed, complete cessation of DMARDs is possible, with drug-free remission (DFR) a well-documented occurrence in 10–20% of patients in longitudinal cohorts [5,6].

Interventional studies of complete DMARD cessation in RA suggest that arthritis flare occurs in approximately half of cases [[7], [8], [9]], a risk that is likely to be unacceptably high for many patients and clinicians owing to the negative impact on quality of life [10,11] and the risk of cumulative joint damage [12,13] with arthritis flare. Prediction of DFR vs. flare prior to DMARD withdrawal would help identify patients in whom DMARD tapering and cessation is more likely to be successful; however, there are currently no reliable biomarkers of DFR to help guide clinicians and patients in this setting.

In this study, we present the findings of a prospective interventional study of complete cessation of conventional synthetic DMARDs in patients with RA in stable remission. Our aim was to identify biomarkers across a broad spectrum of domains – clinical, ultrasound, serological, and transcriptional – which, when measured prior to DMARD cessation, predict future drug-free remission.

## 2 Materials and methods

### 2.1 Recruitment criteria

Eligible patients were identified by their supervising rheumatology clinical team across five National Health Service (NHS) Trusts in the North East of England between September 2014 and October 2016. Patients were eligible for study enrolment if they had a clinical diagnosis of RA made at least 12 months previously and were judged to be currently in clinical remission by their healthcare professional. Only methotrexate, sulfasalazine and/or hydroxychloroquine therapy was permitted; patients receiving biologics or any other DMARD in the past 6 months (or 12 months in the case of leflunomide), or glucocorticoids (enteral, parenteral or intra-articular) in the past 3 months, were excluded. Patients who were part of another clinical trial, and women who were planning pregnancy in the next 6 months, were also excluded.

### 2.2 Study design

In order to be eligible for DMARD cessation, patients had to be in clinical remission at the point of study enrolment with no power Doppler signal on a 7-joint ultrasound examination (see below). Initially, the 2011 ACR/EULAR Boolean remission criteria [14] were used to define remission. However, in order to facilitate recruitment and allow for analysis of baseline ACR/EULAR Boolean remission criteria as a predictor of drug-free clinical remission, this was changed following study amendment approval to a disease activity score in 28 joints with C-reactive protein (DAS28-CRP) < 2.4 [15,16]. Eligible patients completely stopped all DMARD therapy without tapering. All other medications were continued, including non-steroidal anti-inflammatory drugs if required. Routine study reviews were scheduled at month 1, month 3 and month 6, with additional study visits in the case of suspected flare at patient request. The primary outcome was time-to-flare, defined as a DAS28-CRP ≥ 2.4 at any time during the six month follow-up period. A single measure of DAS28-CRP ≥2.4 was permitted if there was an alternative explanation (e.g. concurrent infection causing a rise in inflammatory markers) – in these cases, a repeat DAS28-CRP < 2.4 two weeks later was mandatory for continuation in the study. Patients who experienced an arthritis flare could receive glucocorticoids (parenteral, intra-articular or enteral) at physician discretion, before being discharged from the study to rapidly recommence DMARDs under the guidance of their rheumatologist.

The study design approved by the North East - Tyne & Wear South Research Ethics Committee (National Health Service Health Research Authority, reference 14/NE/1042). The study was conducted in accordance with the Declaration of Helsinki, and all patients provided informed written consent.

### 2.3 Clinical variable assessment

A pre-specified list of clinical variables were recorded, with corroboration of data by clinical notes review (Supplementary Table S1). Serum C-reactive protein (CRP), erythrocyte sedimentation rate (ESR), rheumatoid factor (RhF) titre, and anti-citrullinated peptide autoantibody (ACPA) titre were measured by the hospital clinical laboratory. Where CRP levels fell below the detection threshold of the local laboratory (<5 mg/L), a value of zero was used for the purposes of DAS28-CRP calculation.

### 2.4 Ultrasound (US) assessment

US was performed at study enrolment and month 6 using the same machine (Xario XG Diagnostic Ultrasound System model SSA-680A, Toshiba Medical Systems Corporation) by the same operator (KFB) who is trained in musculoskeletal US assessment. All scans were performed using the same linear mixed array transducer (part number PLT-1204BT). B-mode frequency was fixed at 12 MHz for all scans, and B-mode gain was individually set to a level providing optimal contrast between soft tissue, tendons and bony surfaces, Power Doppler images were acquired at a Doppler frequency of 5.3 MHz for all scans, with Doppler gain individually set to the maximum level possible without cortical bone artefact.

A minimum of 30 still images were recorded per scan, corresponding to the individual views of the seven joints of the US7 protocol of Backhaus et al.: [17] the dominant wrist, 2nd and 3rd metacarpophalangeal joints, 2nd and 3rd proximal interphalangeal joints, and 2nd and 5th metatarsophalangeal joints. Baseline scans were performed blinded to the disease activity score. The level of GS at each joint, and the levels of PD at each joint and tendon complex, were scored using the semi-quantitative scales (0-3) as per the approach of Scheel et al. [18] and Szkudlarek et al. [19] respectively. Tendon-associated GS and joint erosions were scored as either present (1) or absent (0). Minor vessel-related Doppler signal at the wrist was not scored as power Doppler signal so long as all of the following criteria were satisfied: a) only a single vessel was present; and b) the origin of the vessel could be easily visualised as arising from a vessel superficial to the tendons of extensor digitorum; and c) no further branching of the vessel occurred below deep to the tendons of extensor digitorum; and d) the vessel did not traverse any areas of any level of greyscale change. Such an approach is in keeping with representative images from a published atlas of musculoskeletal ultrasonographic scoring for use in clinical research [20]. Scan images were rescored by KFB and a second observer (BT) with good intra- and inter-rater agreement (overall Cohen's kappa 0.73 and 0.62 respectively).

### 2.5 Laboratory procedures

#### 2.5.1 CD4^+^ T cell isolation and RNA extraction

CD4^+^ T cells were isolated from peripheral blood samples by negative CD36 selection followed by positive CD4 selection as previously described [21]. The median (IQR, range) purity of CD4^+^ T cell isolations was 99.0% (98.3–99.3, 95.8–99.7) as confirmed by flow cytometry, with a median (IQR) yield of 2.2 (1.6–2.9) x 10^5^ cells per ml whole blood. Extracted T cells were then immediately lysed in the presence of β-mercaptoethanol before freezing at −80 °C. Frozen T cell lysates were subsequently thawed, and RNA was extracted using the AllPrep™ DNA/RNA/miRNA Universal Kit (Qiagen) as per the manufacturer's instructions. The quantity and quality of RNA in each T cell lysate was measured by gel electrophoresis using a Tapestation™ 4200 machine (Agilent). The median (IQR, range) RNA yield was 838 (636–976, 277–2275) ng per million cells lysed. The quality of RNA was excellent, with a median (IQR, range) estimated RNA integrity number (RIN^e^) of 9.4 (9.1–9.5, 8.7–9.8).

#### 2.5.2 Next-generation RNA sequencing (RNAseq)

1.5 μg of total RNA per sample was used for RNAseq processing; where total RNA < 1.5 μg, the entire sample was used. Total RNA was processed using the TruSeq™ Stranded mRNA Library Prep Kit (Illumina), according to the ‘High Sample Protocol’ section of the manufacturer's instructions. RNAseq was performed using an Illumina NextSeq™ 500 in high-output mode. This configuration delivered 400 million reads over 75 cycles for 40 samples loaded across 4 lanes per flow cell. Sequencing was performed in batches across 4 separate flow cell sequencing runs. Samples were allocated to sequencing batches such that computational correction for any batch-to-batch variation at the level of either the RNA extraction (6 batches) or RNA sequencing (4 batches) could be achieved, according to a predetermined experimental design using the duplicate correlation command of the ‘limma’ Bioconductor/R package (v3.32.5) [22]. Samples were sequenced to a mean (range) depth of 12.1 (9.4–18.4) reads per sample, with excellent quality demonstrated by a mean Phred score >30 across all read positions.

Transcript abundance was estimated from the raw FASTQ files using Kallisto software (v0.43.0) [23] ran in single-end mode, and using an index based on Gencode v24 transcript sequences [24]. Read counts were imported to R (v3.4.1) [25] using the ‘tximport’ package [26], removing genes with mean read count of <60. Gene annotation using the Ensembl GRCh38 assembly [27] was performed using the ‘biomaRt’ package [28]. Read counts were normalised using trimmed mean of M-values normalisation (TMM), and were then logarithmically transformed to log counts per million (logCPM) using the variance modelling at the observational level (voom) approach [29]. CD4^+^ T cell gene expression data are available at the NCBI Gene Expression Omnibus (accession number GSE122612).

#### 2.5.3 Serum protein biomarkers

The levels of 39 circulating cytokines, chemokines and acute phase proteins were measured by electrochemiluminescence (V-PLEX™ plates, MesoScale Discovery) according to the manufacturer's instructions. All baseline samples were processed together on the same plates to avoid batch variation. Assays where <20% of measurements fell above the lower limit of detection were excluded, leaving 26 biomarkers available for analysis (Supplementary Fig. S1). Baseline serum protein biomarker data were unavailable for one patient, who was excluded from the serum and integrative biomarker analyses.

### 2.6 Statistical analysis

This was an exploratory study to identify biomarkers for future validation, and the statistical analyses were conducted in line with this to prioritize the reduction of type II error. Analysis was performed in the R environment, version 3.3.2 [25], with additional packages as specified, according to the following standardised schedule.

First, the association between each variable and time-to-flare was analysed by univariate Cox regression within each variable domain (i.e. clinical, ultrasound, serum protein, RNAseq) using the ‘survival’ package [30]. Next, variables were selected based on their univariate p-value to be taken forward to a multivariate Cox regression model. For clinical, ultrasound and cytokine data, an elevated significance threshold (p < 0.2) was used in order to reduce the risk of type II error at this preliminary stage, in keeping with established precedent [31,32]. A more stringent significance threshold (p < 0.001) was utilised for RNAseq univariate analysis in reflection of the greater number of variables analysed. Variables were then advanced to multivariate Cox regression with backwards stepwise variable selection based on the Akaike information criterion (using the ‘MASS’ package [14]). Variables that remained significantly (p < 0.05, or <0.001 for RNAseq data) associated with time-to-flare in each domain multivariate model were then combined in a final multivariate integrative analysis to form a composite score, weighted by their respective coefficients. No significant departure from proportional hazards (as assessed by Schoenfeld residuals) was observed except where stated. An optimum biomarker threshold based on Youden's index was then calculated using receiver operating characteristic (ROC) analysis to assess the sensitivity/specificity and area under the ROC curve (ROC_AUC_), with 95% confidence intervals calculated using bootstrapping (2000 replicates) and the DeLong procedure respectively (using the ‘pROC’ package [33]). Survival curves were compared between the dichotomised groups (using the ‘survminer’ package [34]) by the log-rank test as a measure of their utility in predicting time-to-flare after DMARD cessation.

## 3 Results

### 3.1 Patient outcomes

**Fig. 1:**
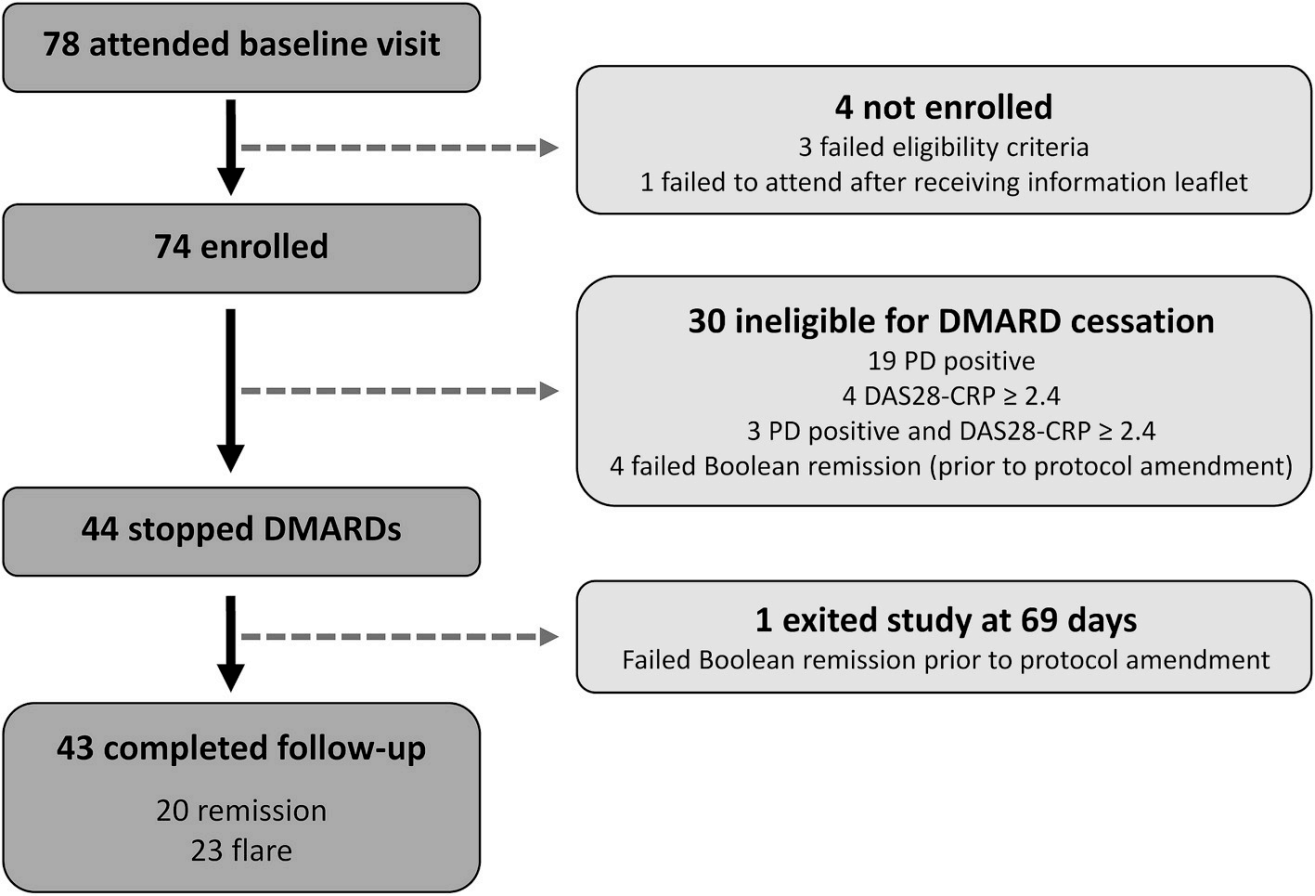
**Study design and recruitment**. 78 patients attended a baseline visit, of whom 44 stopped disease-modifying anti-rheumatic drug (DMARD) therapy. Patients then attended routine study visits at 1, 3 and 6 months following DMARD cessation, with additional unscheduled visits at the request of the patient in the event of suspected arthritis flare. Flare was confirmed if disease activity score in 28 joints with CRP (DAS28-CRP) ≥ 2.4, at which point the patient exited the study to restart DMARD therapy via their referring rheumatology team. Patients who maintained drug-free remission at 6 months remained without DMARDs and exited the study. PD: power Doppler.

78 patients attended for baseline assessment, of which 44 patients were eligible for DMARD cessation (Fig. 1). Prior to revision of the remission criterion by protocol amendment, one patient exited the study at 69 days despite remaining in DAS28-CRP remission, and was censored in remission at this time point. Of the patients who discontinued DMARDs the majority had established but stable disease, all were Caucasian, and all satisfied the 2010 ACR/EULAR RA classification criteria [35] (Table 1).

**Table 1:** Baseline demographics of the patients who stopped disease-modifying anti-rheumatic drugs (DMARDs).

Demographic	Patients who stopped DMARDs (n = 44)
Female: n (%)	23 (52)
Age: median (IQR) [range]	66.5 (54.5–71.3) [35–82]
Years since RA diagnosis: median (IQR) [range]	5.5 (3–11) [1–40]
RhF positive: n (%)	25 (57)
ACPA positive: n (%)	25 (57)
RhF or ACPA positive: n (%)	32 (73)
Presence of joint erosion on ultrasound: n (%)	29 (70)
Swollen (28) joint count: median (IQR) [range]	0 (0–0) [0–2]
Tender (28) joint count: median (IQR) [range]	0 (0–0) [0–2]
Patient VAS (mm): median (IQR) [range]	3 (1–10) [0–35]
CRP in mg/L: median (IQR) [range]	0 (0–0) [0–13]
ESR in mm/hr: median (IQR) [range]	9 (2–15) [1–77]^a^
DAS28-CRP: median (IQR) [range]	1.07 (0.99–1.63) [0.96–2.34]
ACR/EULAR Boolean remission: n (%)	29 (66)
Months since last steroid: median (IQR) [range]	31 (13–48) [4–95]
Months since last change in DMARDs: median (IQR) [range]	22.5 (12–48.5) [2–132]
Total DMARDs since diagnosis: median [range]	2 [1–4]
Current MTX monotherapy: n (%)	23 (52)
Current SFZ monotherapy: n (%)	4 (9)
Current HCQ monotherapy: n (%)	1 (2)
Current MTX+SFZ: n (%)	5 (11)
Current MTX+HCQ: n (%)	8 (18)
Current SFZ+HCQ: n (%)	1 (2)
Current MTX+SFZ+HCQ: n (%)	2 (5)

ACPA: anti-citrullinated peptide antibody; ACR: American College of Rheumatology; CRP: C-reactive protein; ESR: erythrocyte sedimentation rate; EULAR: European League Against Rheumatism; HCQ: hydroxychloroquine; IQR: interquartile range; MTX: methotrexate; RhF: rheumatoid factor; SFZ: sulfasalazine.

a : one patient had an elevated ESR of 77 at baseline due to hypergammaglobulinaemia from secondary Sjögren's syndrome.

**Fig. 2:**
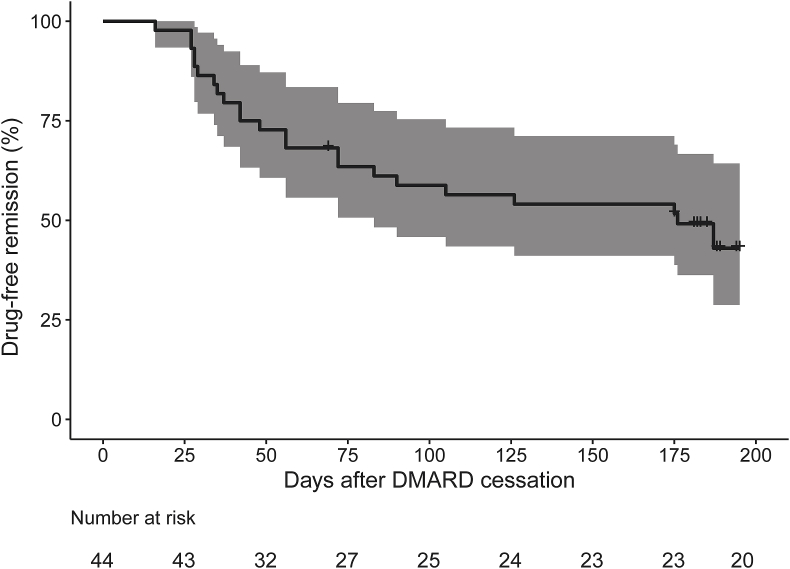
**Kaplan-Meier plot showing maintenance of drug-free remission following disease-modifying anti-rheumatic drug (DMARD) cessation**. Shaded area depicts the 95% confidence interval.

23/44 (52%) patients experienced an arthritis flare at a median (IQR) time to flare of 48 (31.5–86.5) days after DMARD cessation (Fig. 2). The median (IQR, range) DAS28-CRP score at the time of flare was 3.12 (2.62–3.94, 1.58–4.51). One patient was classified as flare despite a DAS28-CRP of 1.58 due to the presence of synovitis (clinical and ultrasound) in the ankles and feet – discounting this patient gives a DAS28-CRP range of 2.45–4.51 at the time of flare. A further patient (who maintained DFR) was treated with a 7 day course of oral prednisolone by their general practitioner for nasal polyposis at 5 months after DMARD cessation; no other patients received systemic steroids during the course of the study.

There were no breaches of study protocol. There were 101 adverse events recorded, none of which were judged to be a consequence of DMARD cessation (Supplementary Table S2). There were no serious adverse events.

### 3.2 Clinical biomarkers

The association between baseline clinical variables and time-to-flare was assessed by univariate Cox regression (Supplementary Table S3). Those with a univariate p < 0.2 were advanced to form a multivariate stepwise Cox model incorporating 9 baseline clinical variables, of which 6 were associated with time-to-flare at the p < 0.05 significance level (Table 2). RhF positivity, longer time from diagnosis to starting first DMARD, and longer symptom duration at time of diagnosis were all associated with an increased hazard of flare. In contrast, fulfilment of ACR/EULAR Boolean remission criteria at baseline, longer time since last change in DMARD therapy, and longer disease duration were associated with a reduced hazard of flare.

**Table 2:** Association of clinical variables with occurrence of arthritis flare following disease-modifying anti-rheumatic drug (DMARD) cessation, using a backwards stepwise multivariate Cox regression model.

Variable	B	HR_flare_	95% CI	p
RhF positive	1.839	6.29	1.61–24.53	0.008
Months since last change in DMARD therapy	−0.030	0.97	0.95–0.99	0.014
ACR/EULAR Boolean remission	−1.330	0.26	0.09–0.77	0.014
Months from first rheumatology review to starting first DMARD	0.034	1.03	1.01–1.06	0.015
Symptom duration prior to first rheumatology review (months)	0.063	1.06	1.01–1.12	0.025
Disease duration (years)	−0.155	0.86	0.74–1.00	0.043
Current methotrexate	2.261	9.59	0.99–93.29	0.051
Weekly alcohol unit intake	0.050	1.05	0.99–1.11	0.090
ACPA positive	1.078	2.94	0.69–12.48	0.144

For continuous variables, hazard ratios (HR) and the Cox regression coefficients (B) are presented for a 1-unit change in that variable. Statistical significance calculated by the Wald test. ACPA: anti-citrullinated peptide antibody; ACR: American College of Rheumatology; CI: confidence interval; EULAR: European League Against Rheumatism; RhF: rheumatoid factor.

### 3.3 Ultrasound biomarkers

Total greyscale synovial, greyscale tenosynovial, and joint erosion scores were not significantly associated with time-to-flare in univariate Cox regression analysis (Supplementary Table S4).

### 3.4 Serum protein biomarkers

Ten serum protein biomarkers were associated with time-to-flare in univariate Cox regression analyses at a p < 0.2 significance threshold (Supplementary Table S5). These variables were advanced to multivariate Cox regression analysis, and after backwards stepwise selection reduced to three biomarkers, two of which retained an association (p < 0.05) with time-to-flare: monocyte chemoattractant protein 1 (MCP1) (HR_flare_ 10.2, 95% CI 2.01–51.4, p = 0.005) and interleukin-27 (IL-27) (HR_flare_ 4.32, 95% CI 1.17–16.0, p = 0.029).

### 3.5 CD4^+^ T cell RNAseq biomarkers

The baseline expression of 19 genes within peripheral CD4^+^ T cells was associated with time-to-flare in univariate Cox regression analyses at the p < 0.001 significance threshold (Supplementary Table S6). From these genes, a multivariate stepwise Cox regression model was formed incorporating 11 genes, of which three were significant at the p < 0.001 threshold (Supplementary Table S7). Two of these genes were associated with an increased hazard ratio (HR) of flare: family with sequence similarity 102 member B (*FAM102B;* HR_flare_ 1060, 95% CI 22.6–50000, p = 3.88 x 10^−4^) and the predicted novel antisense gene *ENSG00000227070* (HR_flare_ 5.94, 95% CI 2.08–16.9, p = 8.63 x 10^−4^). In contrast, the remaining gene (*ENSG00000228010*, also a predicted novel antisense gene) was associated with increased chance of sustained drug-free remission (HR_flare_ 0.02, 95% CI 0.00–0.14, p = 2.24 x 10^−4^).

### 3.6 Integrative biomarker analysis

Based on the aforementioned multivariate analyses, 11 baseline variables were advanced to a final integrative analysis: six clinical variables (RhF status, ACR/EULAR Boolean remission, months since last change in DMARD therapy, symptom duration at diagnosis, disease duration), two cytokines/chemokines (IL-27, MCP1), and three CD4^+^ T cell genes (*FAM102B*, *ENSG00000227070*, *ENSG00000228010*). In a multivariate backwards stepwise Cox regression model, there was some evidence for departure from the proportional hazards assumption attributable to minor outlying data for ACR/EULAR Boolean remission only, although not for the model as a whole (p = 0.36). Five variables were significantly (p < 0.05) associated with time-to-flare (Table 3), and were combined with their respective coefficients to form a composite biomarker score:

$$\begin{array}{l} \text{Composite}\;\text{biomarker}\;\text{score}=1.08\left(ENSG00000227070\right)+2.90\left(FAM102B\right)+2.13\left(\text{ln}\left[\text{IL}27+1\right]\right)\\ -1.97\left(ENSG00000228010\right)-1.45\left(\text{ACR}/\text{EULAR}\;\text{Boolean}\;\text{remission}\right) \end{array}$$

**Table 3:** Association of baseline variables across all domains with time-to-flare following DMARD-cessation in a backward stepwise multivariate Cox regression model.

Variable	B	HR_flare_	95% CI	p
*ENSG00000228010*	−1.972	0.14	0.05–0.37	<0.001
*ENSG00000227070*	1.080	2.95	1.73–5.01	<0.001
ACR/EULAR Boolean remission	−1.446	0.24	0.09–0.61	0.003
ln(IL27 + 1)	2.131	8.43	2.06–34.48	0.003
*FAM102B*	2.901	18.19	2.26–146.57	0.006
RhF positive	0.729	2.07	0.77–5.60	0.151

For continuous variables, hazard ratios (HR) and the Cox regression coefficients (B) are presented for a 1-unit change in that variable. Statistical significance calculated by the Wald test. ACR: American College of Rheumatology; CI: confidence interval; EULAR: European League Against Rheumatism; *FAM102B*: family with sequence similarity 102 member B IL: interleukin; RhF: rheumatoid factor.

**Fig. 3:**
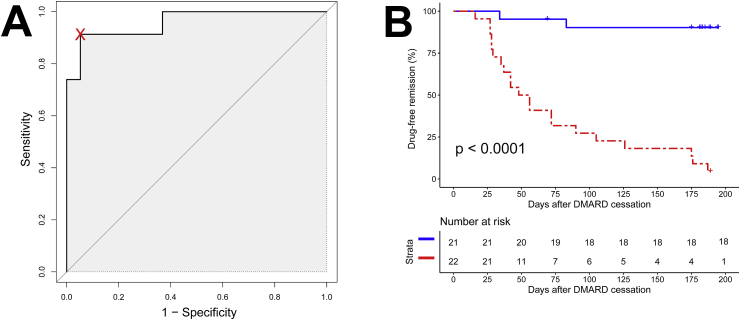
**Prediction of flare/remission using the composite biomarker score.** (A): Receiver-operating characteristic curve for prediction of flare by the composite biomarker. The threshold for a positive versus negative test (39.65) is shown by the cross. (B): Kaplan-Meier plot of maintenance of drug-free remission stratified by positive (red) or negative (blue) baseline composite biomarker score. A significant difference in drug-free remission between the strata was observed (log-rank test). (For interpretation of the references to colour in this figure legend, the reader is referred to the Web version of this article.)

ROC analysis was used to set an optimum threshold (39.65) for the prediction of flare following DMARD cessation (Fig. 3A). The composite biomarker score performed well in predicting arthritis flare, with a sensitivity of 0.91 (95% CI 0.78–1.00), specificity of 0.95 (0.84–1.00), positive predictive value of 0.96 (0.86–1.00), negative predictive value of 0.90 (0.78–1.00), and ROC_AUC_ of 0.96 (0.91–1.00). A negative composite biomarker score (<39.65) was a strong predictor of sustained DMARD-free remission, with a significant difference in DMARD-free survival between those with positive versus negative baseline scores (p < 0.0001, log-rank test) (Fig. 3B).

To account for reclassification of one patient on grounds of ankle/feet flare (see Results 3.1 above), a sensitivity analysis was performed with this patient classified in remission with no notable effect on biomarker performance (Supplementary Tables S8 and S9).

## 4 Discussion

Advances in the management of RA over recent years have made sustained remission a realistic and achievable goal for many patients. This has, however, ushered a new dilemma into the clinic: how best to manage potentially toxic and costly DMARD therapy in such individuals. There have been several recently published studies of DMARD tapering in RA remission [36], although the majority focus on the partial tapering or spacing of biologic agents with continuation of conventional synthetic DMARDs, rather than addressing the concept of complete DMARD cessation.

Our unique study design of abrupt and complete DMARD cessation enabled us to compare baseline characteristics in subsequently flaring and non-flaring patients. 21/44 (48%) patients maintained clinical remission for 6 months following DMARD cessation, an observation that is comparable with previously published studies. In the RETRO study, randomisation to withdrawal (tapering followed by cessation, or immediate cessation) of a variety of biologics and conventional synthetic DMARDs resulted in DFR (DAS28-ESR < 2.6) in 35/63 (56%) patients at 12 months, compared to sustained remission in 32/38 (84%) patients who continued DMARD therapy [8]. In the BeSt study, DFR (DAS44 < 1.6) was observed in 59/115 (51%) patients who tapered DMARDs to complete cessation, with a median duration of remission of 23 months [7]. The consistent rate of DFR observed across these studies is remarkable given the heterogeneity of DMARD therapy, and perhaps suggests an intrinsic propensity for DFR within disease subtypes that may be independent of the specific DMARDs initially used to achieve remission [37]. In this context the relationship between drug-free remission and true immune tolerance deserves further exploration, particularly because there is a high unmet need for biomarkers to guide tolerogenic therapy development and implementation [38].

Lower disease activity at the point of DMARD cessation/tapering was predictive of DFR in several previous studies [5,7,39], in keeping with the predictive value of ACR/EULAR Boolean remission observed in our study. Interestingly, neither its individual components (i.e. swollen/tender joint counts, patient global assessment) nor DAS28-CRP demonstrated predictive value in our study. In contrast, ACR/EULAR Boolean remission was not predictive of future DFR in the RETRO study [8]. It is possible that modifications to the ACR/EULAR Boolean construct, such as a relaxation of the patient global assessment threshold (which has been criticised by some as overly strict [[40], [41], [42], [43], [44], [45], [46]]), may improve its predictive utility in this setting; however, our limited sample size hinders further exploration.

It is possible that a longer duration of sustained remission prior to DMARD withdrawal may favour successful achievement of DFR. Higher rates of DFR are indeed observed with the use of modern treat-to-target DMARD regimens (where clinical remission is more likely to be achieved early in the course of disease) compared to historical treatment approaches [6], supporting this assumption. Furthermore, longer duration of DAS-defined remission was associated with higher rates of DFR following withdrawal of abatacept in the AVERT study [47], and lower mean disease activity prior to DMARD withdrawal was predictive of DFR in BeST [7]. In our study, the lack of a lead-in monitoring period before DMARD cessation prohibits a direct analysis of the value of remission duration in predicting DFR. Nevertheless, an indirect measure of remission duration – namely time since last change in DMARD therapy – is positively associated with achieving DFR, albeit at an insufficient magnitude to advance to the final integrative biomarker score.

Seronegativity for ACPA and RhF have previously been shown to be predictive of DFR [5,[7], [8], [9],^48^], as observed for RhF in the clinical biomarker analysis of our study. In the RETRO study, combination of ACPA with the 12-cytokine multibiomarker disease activity (MBDA) score [49] further increased its ability to predict DFR vs. flare following DMARD cessation [50]. However, in our study ACPA and/or RhF status did not provide any additional predictive value beyond the five variables in the final composite biomarker score. We furthermore observe that IL-27 is associated with increased risk of flare following DMARD cessation. Indeed, IL-27 has been implicated in the pathogenesis of RA [[51], [52], [53], [54], [55]], though has also shown protective effects against experimentally-induced arthritis in murine models [56,57]. Our results suggest that further exploration of the mechanistic role of IL-27 in the context of arthritis flare may prove valuable.

Our composite biomarker score incorporates the expression of three genes within peripheral CD4^+^ T cells. The function of the FAM102B protein is unknown, although the paralogous FAM102A is known to be involved in oestrogen signalling [58], osteoclast differentiation [59], and cell membrane trafficking [60]. ENSG00000228010 is an antisense RNA gene to zinc finger 12 (*ZNF12)*, a member of the Krüppel C2H2-type zinc finger family with evolutionarily-conserved function in the regulation of developmental gene expression [61]. Interestingly, *ZNF1*2 has been implicated as a causative gene in a quantitative trait locus influencing TNF-α production *in vitro* by human peripheral blood mononuclear cells in response to *Candida albicans* [62], supporting an immunomodulatory role of the gene. ENSG00000227070 is predicted to be a novel antisense RNA gene, though no published data exists as to its putative target (Ensembl genome browser release 95) [63]. To our knowledge, only one other study has explored differential gene expression within peripheral CD4^+^ T cells in the context of DFR in RA. However, this exploratory analysis of the U-Act-Early study focussed on differential gene expression at the time of disease diagnosis using a network analytic approach [64], thus limiting a direct comparison with our results.

A striking observation is the lack of association of ultrasound biomarkers with patient outcome following DMARD cessation. However, to alleviate any potential concerns of referring clinicians, patients with any degree of power Doppler signal were excluded from DMARD cessation, thus preventing an assessment of this important ultrasound parameter. Furthermore, significant abnormalities may have been present outside of the seven joints included within the US7 scan. Nevertheless, a lack of predictive value of ultrasound in DMARD tapering and cessation was also observed by El Miedany et al. [48], who found no association between future flare and either greyscale or power Doppler abnormalities in an extended 40-joint scan protocol.

There are several limitations to this study, notably its small size, short duration of follow-up, and heterogeneity of DMARDs at enrolment. Over-fitting of the data is likely given that the number of candidate variables is greater than the number of study participants, and the impressive biomarker performance presented herein needs to be interpreted within this context. Indeed, it is now a priority to validate our findings in an external cohort, a crucial next step before considering application to clinical practice.

## 5 Conclusions

In summary, we describe the integration of variables across multiple domains (clinical, ultrasound, serological, gene expression) at an unprecedented resolution to predict DFR in RA. A composite biomarker score, based on only five baseline variables measured before DMARD cessation, had excellent predictive value for DFR at 6 months. If successfully validated in an external cohort, our biomarker score would hold promise in identifying those patients for whom drug withdrawal is appropriate, thus guiding an intelligent and personalised approach to DMARD therapy in RA remission.

## Conflicts of interest

KFB, JDI, AGP and DWL are named as inventors on a patent application by Newcastle University relating to the prediction of drug-free remission in rheumatoid arthritis based on the results of this study. BT, AJS and AS have no conflicts of interest to declare.

## Funding

This study was funded by a Wellcome Trust Translational Medicine and Therapeutics Clinical PhD Fellowship (102595/Z/13/A to KFB; https://wellcome.ac.uk/), and by a National Institute for Health Research (NIHR) Infrastructure Doctoral Traineeship Award from the Newcastle NIHR Biomedical Research Centre (BH136167/PD0045 to KFB; https://www.nihr.ac.uk/). The funders had no role in the study design, data collection and analysis, decision to publish, or preparation of the manuscript.
