# Erratum to “Predicting drug-free remission in rheumatoid arthritis: A prospective interventional cohort study” [J. Autoimmun. 105 (December 2019) 102298]

**DOI:** 10.1016/j.jaut.2022.102913

**Published:** 2022-10

**Authors:** Kenneth F. Baker, Andrew J. Skelton, Dennis W. Lendrem, Adam Scadeng, Ben Thompson, Arthur G. Pratt, John D. Isaacs

**Affiliations:** a Musculoskeletal Research Group, Institute of Cellular Medicine, Newcastle University, Newcastle upon Tyne, United Kingdom; b Musculoskeletal Unit, Newcastle upon Tyne Hospitals NHS Foundation Trust, Newcastle upon Tyne, United Kingdom; c Bioinformatics Support Unit, Newcastle University, Newcastle upon Tyne, United Kingdom

The publisher regrets that the final pages and a figure were missing from the online PDF document. These are available to view below.

The publisher would like to apologise for any inconvenience caused.

**Figure:**
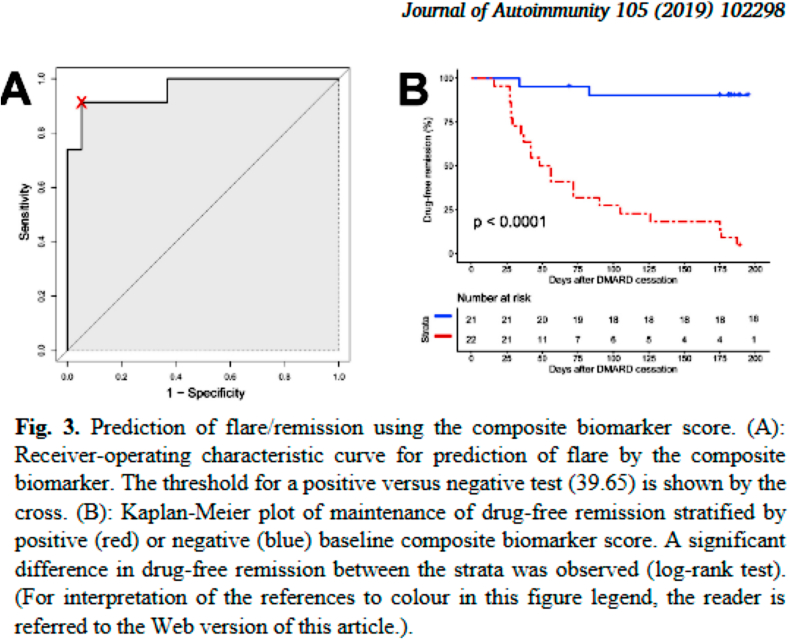

our study. Interestingly, neither its individual components (i.e. swollen/tender joint counts, patient global assessment) nor DAS28-CRP demonstrated predictive value in our study. In contrast, ACR/EULAR Boolean remission was not predictive of future DFR in the RETRO study [8]. It is possible that modifications to the ACR/EULAR Boolean construct, such as a relaxation of the patient global assessment threshold (which has been criticised by some as overly strict [40],[41],[42],[43],[44],[45],[46]), may improve its predictive utility in this setting; however, our limited sample size hinders further exploration.

It is possible that a longer duration of sustained remission prior to DMARD withdrawal may favour successful achievement of DFR. Higher rates of DFR are indeed observed with the use of modern treat-to-target DMARD regimens (where clinical remission is more likely to be achieved early in the course of disease) compared to historical treatment approaches [6], supporting this assumption. Furthermore, longer duration of DAS-defined remission was associated with higher rates of DFR following withdrawal of abatacept in the AVERT study [47], and lower mean disease activity prior to DMARD withdrawal was predictive of DFR in BeST [7]. In our study, the lack of a lead-in monitoring period before DMARD cessation prohibits a direct analysis of the value of remission duration in predicting DFR. Nevertheless, an indirect measure of remission duration – namely time since last change in DMARD therapy – is positively associated with achieving DFR, albeit at an insufficient magnitude to advance to the final integrative biomarker score.

Seronegativity for ACPA and RhF have previously been shown to be predictive of DFR [5],[7],[8],[9],[48], as observed for RhF in the clinical biomarker analysis of our study. In the RETRO study, combination of ACPA with the 12-cytokine multibiomarker disease activity (MBDA) score [49] further increased its ability to predict DFR vs. flare following DMARD cessation [50]. However, in our study ACPA and/or RhF status did not provide any additional predictive value beyond the five variables in the final composite biomarker score. We furthermore observe that IL-27 is associated with increased risk of flare following DMARD cessation. Indeed, IL-27 has been implicated in the pathogenesis of RA [51],[52],[53],[54],[55], though has also shown protective effects against experimentally-induced arthritis in murine models [56,57]. Our results suggest that further exploration of the mechanistic role of IL-27 in the context of arthritis flare may prove valuable.

Our composite biomarker score incorporates the expression of three genes within peripheral CD4^+^ T cells. The function of the FAM102B protein is unknown, although the paralogous FAM102A is known to be involved in oestrogen signalling [58], osteoclast differentiation [59], and cell membrane trafficking [60]. ENSG00000228010 is an antisense RNA gene to zinc finger 12 (*ZNF12*), a member of the Krüppel C2H2-type zinc finger family with evolutionarily-conserved function in the regulation of developmental gene expression [61]. Interestingly, *ZNF1*2 has been implicated as a causative gene in a quantitative trait locus influencing TNF-α production *in vitro* by human peripheral blood mononuclear cells in response to *Candida albicans* [62], supporting an immunomodulatory role of the gene. ENSG00000227070 is predicted to be a novel antisense RNA gene, though no published data exists as to its putative target (Ensembl genome browser release 95) [63]. To our knowledge, only one other study has explored differential gene expression within peripheral CD4^+^ T cells in the context of DFR in RA. However, this exploratory analysis of the U-Act-Early study focussed on differential gene expression at the time of disease diagnosis using a network analytic approach [64], thus limiting a direct comparison with our results.

A striking observation is the lack of association of ultrasound biomarkers with patient outcome following DMARD cessation. However, to alleviate any potential concerns of referring clinicians, patients with any degree of power Doppler signal were excluded from DMARD cessation, thus preventing an assessment of this important ultrasound parameter. Furthermore, significant abnormalities may have been present outside of the seven joints included within the US7 scan. Nevertheless, a lack of predictive value of ultrasound in DMARD tapering and cessation was also observed by El Miedany et al. [48], who found no association between future flare and either greyscale or power Doppler abnormalities in an extended 40-joint scan protocol.

There are several limitations to this study, notably its small size, short duration of follow-up, and heterogeneity of DMARDs at enrolment. Over-fitting of the data is likely given that the number of candidate variables is greater than the number of study participants, and the impressive biomarker performance presented herein needs to be interpreted within this context. Indeed, it is now a priority to validate our findings in an external cohort, a crucial next step before considering application to clinical practice.

5. Conclusions

In summary, we describe the integration of variables across multiple domains (clinical, ultrasound, serological, gene expression) at an unprecedented resolution to predict DFR in RA. A composite biomarker score, based on only five baseline variables measured before DMARD cessation, had excellent predictive value for DFR at 6 months. If successfully validated in an external cohort, our biomarker score would hold promise in identifying those patients for whom drug withdrawal is appropriate, thus guiding an intelligent and personalised approach to DMARD therapy in RA remission.

Conflicts of interest

KFB, JDI, AGP and DWL are named as inventors on a patent application by Newcastle University relating to the prediction of drug-free remission in rheumatoid arthritis based on the results of this study. BT, AJS and AS have no conflicts of interest to declare.

Funding

This study was funded by a Wellcome Trust Translational Medicine and Therapeutics Clinical PhD Fellowship (102595/Z/13/A to KFB; https://wellcome.ac.uk/), and by a National Institute for Health Research (NIHR) Infrastructure Doctoral Traineeship Award from the Newcastle NIHR Biomedical Research Centre (BH136167/PD0045 to KFB; https://www.nihr.ac.uk/). The funders had no role in the study design, data collection and analysis, decision to publish, or preparation of the manuscript.

Acknowledgements

We thank all of the patients who participated in this study, and all of the rheumatology health professionals who referred patients to the study. We also thank Oliver Eltherington, Nicola Maney, Laura Ridgley and Natasha West for their assistance with laboratory processing. We acknowledge the Flow Cytometry Core Facility and the Genomics Core Facility at Newcastle University for assistance with the generation of flow cytometry and RNAseq data respectively. Results from this study were previously presented at the EULAR 2018 Congress [65], and form the basis of a PhD Thesis (Newcastle University) by KFB. The research was supported by the National Institute for Health Research Newcastle Biomedical Research Centre based at Newcastle Hospitals NHS Foundation Trust and Newcastle University. The views expressed are those of the authors and not necessarily those of the NHS, the NIHR or the Department of Health.

Appendix A. Supplementary data

The following are the Supplementary data to this article:

Multimedia component 1.

References

[1] J.S. Smolen, R. Landewé, J. Bijlsma, G. Burmester, K. Chatzidionysiou, M. Dougados et al., EULAR recommendations for the management of rheumatoid arthritis with synthetic and biological disease-modifying antirheumatic drugs: 2016 update. Ann. Rheum. Dis. 76 (2017), p. 960.

[2] J.S. Smolen, F.C. Breedveld, G.R. Burmester, V. Bykerk, M. Dougados, P. Emery et al., Treating rheumatoid arthritis to target: 2014 update of the recommendations of an international task force. Ann. Rheum. Dis. 75 (2016), pp. 3-15.

[3] J. Ledingham, N. Gullick, K. Irving, R. Gorodkin, M. Aris, J. Burke et al., BSR and BHPR guideline for the prescription and monitoring of non-biologic disease-modifying anti-rheumatic drugs. Rheumatology, 56 (2017), pp. 865-868.

[4] J.A. Singh, K.G. Saag, S.L. Bridges Jr. E.A. Akl, R.R. Bannuru, M.C. Sullivan et al., 2015 American College of rheumatology guideline for the treatment of rheumatoid arthritis. Arthritis Rheum. 68 (2016), pp. 1–26.

[5] D. van der Woude, A. Young, K. Jayakumar, B.J. Mertens, R.E. Toes, D. van der Heijde et al., Prevalence of and predictive factors for sustained disease-modifying antirheumatic drug-free remission in rheumatoid arthritis: Results from two large early arthritis cohorts. Arthritis Rheum. 60 (2009), pp. 2262–2271.

[6] S. Ajeganova, H.W. van Steenbergen, J.A. van Nies, L.E. Burgers, T.W. Huizinga, A.H. van der Helm-van Mil. Disease-modifying antirheumatic drug-free sustained remission in rheumatoid arthritis: An increasingly achievable outcome with subsidence of disease symptoms. Ann. Rheum. Dis. 75 (2016), pp. 867–873.

[7] N.B. Klarenbeek, S.M. van der Kooij, M. Guler-Yuksel, J.H. van Groenendael, K.H. Han, P.J. Kerstens et al. Discontinuing treatment in patients with rheumatoid arthritis in sustained clinical remission: Exploratory analyses from the BeSt study. Ann. Rheum. Dis. 70 (2011), pp. 315–319.

[8] J. Haschka, M. Englbrecht, A.J. Hueber, B. Manger, A. Kleyer, M. Reiser et al. Relapse rates in patients with rheumatoid arthritis in stable remission tapering or stopping antirheumatic therapy: Interim results from the prospective randomised controlled RETRO study. Ann. Rheum. Dis. 75 (2016), pp. 45–51.

[9] S. ten Wolde, F.C. Breedveld, J. Hermans, J.P. Vandenbroucke, M.A. van de Laar, H.M. Markusse et al. Randomised placebo-controlled study of stopping second-line drugs in rheumatoid arthritis. Lancet, 347 (1996), pp. 347–352.

[10] C.A. Flurey, M. Morris, P. Richards, R. Hughes, S. Hewlett. It's like a juggling act: Rheumatoid arthritis patient perspectives on daily life and flare while on current treatment regimes. Rheumatology, 53 (2014), pp. 696–703.

[11] S. Hewlett, T. Sanderson, J. May, R. Alten, C.O. Bingham 3rd, M. Cross et al. ‘I'm hurting, I want to kill myself’: Rheumatoid arthritis flare is more than a high joint count--an international patient perspective on flare where medical help is sought. Rheumatology, 51 (2012), pp. 69–76.

[12] I.M. Markusse, L. Dirven, A.H. Gerards, J.H. van Groenendael, H.K. Ronday, P.J. Kerstens et al. Disease flares in rheumatoid arthritis are associated with joint damage progression and disability: 10-year results from the BeSt study. Arthritis Res. Ther. 17 (2015), p. 232.

[13] F. Ometto, B. Raffeiner, L. Bernardi, C. Botsios, N. Veronese, L. Punzi et al. Self-reported flares are predictors of radiographic progression in rheumatoid arthritis patients in 28-joint disease activity score remission: A 24-month observational study. Arthritis Res. Ther. 18 (2016), p. 89.

[14] D.T. Felson, J.S. Smolen, G. Wells, B. Zhang, L.H. van Tuyl, J. Funovits et al. American College of Rheumatology/European League against Rheumatism provisional definition of remission in rheumatoid arthritis for clinical trials. Arthritis Rheum. 63 (2011), pp. 573–586.

[15] J. Fransen, P.M.J. Welsing, R.M.H. De Keijzer, P.L.C.M. van Riel. Development and validation of the DAS28 using CRP. Ann. Rheum. Dis. 62 (2003), p. 10.

[16] R. Fleischmann, D. van der Heijde, A.S. Koenig, R. Pedersen, A. Szumski, L. Marshall et al. How much does Disease Activity Score in 28 joints ESR and CRP calculations underestimate disease activity compared with the Simplified Disease Activity Index? Ann. Rheum. Dis. 74 (2015), pp. 1132–1137.

[17] M. Backhaus, S. Ohrndorf, H. Kellner, J. Strunk, T.M. Backhaus, W. Hartung et al. Evaluation of a novel 7-joint ultrasound score in daily rheumatologic practice: A pilot project. Arthritis Rheum. 61 (2009), pp. 1194–1201.

[18] A.K. Scheel, K.G. Hermann, E. Kahler, D. Pasewaldt, J. Fritz, B. Hamm et al. A novel ultrasonographic synovitis scoring system suitable for analyzing finger joint inflammation in rheumatoid arthritis. Arthritis Rheum. 52 (2005), pp. 733–743.

[19] M. Szkudlarek, M. Court-Payen, S. Jacobsen, M. Klarlund, H.S. Thomsen, M. Ostergaard. Interobserver agreement in ultrasonography of the finger and toe joints in rheumatoid arthritis. Arthritis Rheum. 48 (2003), pp. 955–962.

[20] H.B. Hammer, P. Bolton-King, V. Bakkeheim, T.H. Berg, E. Sundt, A.K. Kongtorp et al. Examination of intra and interrater reliability with a new ultrasonographic reference atlas for scoring of synovitis in patients with rheumatoid arthritis. Ann. Rheum. Dis. 70 (2011), pp. 1995–1998.

[21] A.G. Pratt, D.C. Swan, S. Richardson, G. Wilson, C.M. Hilkens, D.A. Young et al. A CD4 T cell gene signature for early rheumatoid arthritis implicates interleukin 6-mediated STAT3 signalling, particularly in anti-citrullinated peptide antibody-negative disease. Ann. Rheum. Dis. 71 (2012), pp. 1374–1381.

[22] M.E. Ritchie, B. Phipson, D. Wu, Y. Hu, C.W. Law, W. Shi et al. Limma powers differential expression analyses for RNA-sequencing and microarray studies. Nucleic Acids Res. 43 (2015), p. e47.

[23] N.L. Bray, H. Pimentel, P. Melsted, L. Pachter. Near-optimal probabilistic RNA-seq quantification. Nat. Biotechnol. 34 (2016), pp. 525–527.

[24] J. Harrow, A. Frankish, J.M. Gonzalez, E. Tapanari, M. Diekhans, F. Kokocinski et al. GENCODE: The reference human genome annotation for the ENCODE Project. Genome Res. 22 (2012), pp. 1760–1774.

[25] R Core Team. R. A Language and Environment for Statistical Computing. R Foundation for Statistical Computing, Vienna, Austria (2016). https://www.R-project.org/, Accessed 27th Mar 2019.

[26] C. Soneson, M.I. Love, M.D. Robinson. Differential analyses for RNA-seq: Transcript-level estimates improve gene-level inferences. F1000Res, 4 (2015), p. 1521.

[27] A. Yates, W. Akanni, M.R. Amode, D. Barrell, K. Billis, D. Carvalho-Silva et al. Ensembl 2016. Nucleic Acids Res. 44 (2016), pp. D710-D716.

[28] S. Durinck, P.T. Spellman, E. Birney, W. Huber. Mapping identifiers for the integration of genomic datasets with the R/Bioconductor package biomaRt. Nat. Protoc. 4 (2009), pp. 1184–1191.

[29] C.W. Law, Y. Chen, W. Shi, G.K. Smyth. voom: Precision weights unlock linear model analysis tools for RNA-seq read counts. Genome Biol. 15 (2014), p. R29.

[30] T. Therneau. A package for survival analysis in S. version 2.38. https://CRAN.R-project.org/package=survival (2015), Accessed 27th Mar 2019.

[31] R.M. Mickey, S. Greenland. The impact of confounder selection criteria on effect estimation. Am. J. Epidemiol. 129 (1989), pp. 125–137.

[32] L.G. Dales, H.K. Ury. An improper use of statistical significance testing in studying covariables. Int. J. Epidemiol. 7 (1978), pp. 373–375.

[33] X. Robin, N. Turck, A. Hainard, N. Tiberti, F. Lisacek, J.C. Sanchez et al. pROC: An open-source package for R and S+ to analyze and compare ROC curves. BMC Bioinf. 12 (2011), p. 77.

[34] A. Kassambara, M. Kosinski survminer. Drawing survival curves using ‘ggplot2’. R package version 0.4.3. https://CRAN.R-project.org/package=survminer (2018), Accessed 27th Mar 2019.

[35] D. Aletaha, T. Neogi, A.J. Silman, J. Funovits, D.T. Felson, C.O. Bingham 3rd et al., 2010 Rheumatoid arthritis classification criteria: An American College of Rheumatology/European League against Rheumatism collaborative initiative. Arthritis Rheum. 62 (2010), pp. 2569–2581.

[36] G. Schett, P. Emery, Y. Tanaka, G. Burmester, D.S. Pisetsky, E. Naredo et al. Tapering biologic and conventional DMARD therapy in rheumatoid arthritis: Current evidence and future directions. Ann. Rheum. Dis. 75 (2016), pp. 1428–1437.

[37] I.C. Scott, G.H. Kingsley, D.L. Scott. Can we discontinue synthetic disease-modifying anti-rheumatic drugs in rheumatoid arthritis? Clin. Exp. Rheumatol. 31 (2013), pp. S4–S8.

[38] F. Rayner, J.D. Isaacs. Therapeutic tolerance in autoimmune disease. Semin. Arthritis Rheum. 48 (2018), pp. 558–562.

[39] T.M. Kuijper, J.J. Luime, P.H. de Jong, A.H. Gerards, D. van Zeben, I. Tchetverikov et al. Tapering conventional synthetic DMARDs in patients with early arthritis in sustained remission: 2-year follow-up of the tREACH trial. Ann. Rheum. Dis. 75 (2016), pp. 2119–2123.

[40] B. Kuriya, Y. Sun, G. Boire, B. Haraoui, C. Hitchon, J.E. Pope et al. Remission in early rheumatoid arthritis -- a comparison of new ACR/EULAR remission criteria to established criteria. J. Rheumatol. 39 (2012), pp. 1155–1158.

[41] K.R. Masri, T.S. Shaver, S.H. Shahouri, S. Wang, J.D. Anderson, R.E. Busch et al. Validity and reliability problems with patient global as a component of the ACR/EULAR remission criteria as used in clinical practice. J. Rheumatol. 39 (2012), pp. 1139–1145.

[42] P. Studenic, J.S. Smolen, D. Aletaha. Near misses of ACR/EULAR criteria for remission: Effects of patient global assessment in Boolean and index-based definitions. Ann. Rheum. Dis. 71 (2012), pp. 1702–1705.

[43] M. Vermeer, H.H. Kuper, A.E. van der Bijl, H. Baan, M.D. Posthumus, H.L. Brus et al. The provisional ACR/EULAR definition of remission in RA: A comment on the patient global assessment criterion. Rheumatology, 51 (2012), pp. 1076–1080.

[44] B. Svensson, M.L. Andersson, S.V. Bala, K. Forslind, I. Hafstrom. Long-term sustained remission in a cohort study of patients with rheumatoid arthritis: Choice of remission criteria. BMJ Open, 3 (2013), Article e003554.

[45] K. Thiele, D. Huscher, S. Bischoff, S. Späthling-Mestekemper, M. Backhaus, M. Aringer et al. Performance of the 2011 ACR/EULAR preliminary remission criteria compared with DAS28 remission in unselected patients with rheumatoid arthritis. Ann. Rheum. Dis. 72 (2013), pp. 1194–1199.

[46] K.F. Baker, A.G. Pratt, B. Thompson, J.D. Isaacs. Let's not fool ourselves RA, the ACR/EULAR Remission Criteria Are Not Perfect! Ann Rheum Dis, vol. 76 (2017), p. e12.

[47] P. Emery, G.R. Burmester, V.P. Bykerk, B.G. Combe, D.E. Furst, E. Barre et al. Evaluating drug-free remission with abatacept in early rheumatoid arthritis: Results from the phase 3b, multicentre, randomised, active-controlled AVERT study of 24 months, with a 12-month, double-blind treatment period. Ann. Rheum. Dis. 74 (2015), pp. 19–26.

[48] Y. El Miedany, M. El Gaafary, S. Youssef, I. Ahmed, S. Bahlas, M. Hegazi et al. Optimizing therapy in inflammatory arthritis: Prediction of relapse after tapering or stopping treatment for rheumatoid arthritis patients achieving clinical and radiological remission. Clin. Rheumatol. 35 (2016), pp. 2915–2923.

[49] M. Centola, G. Cavet, Y. Shen, S. Ramanujan, N. Knowlton, K.A. Swan et al. Development of a multi-biomarker disease activity test for rheumatoid arthritis. PLoS One, 8 (2013), Article e60635.

[50] J. Rech, A.J. Hueber, S. Finzel, M. Englbrecht, J. Haschka, B. Manger et al. Prediction of disease relapses by multibiomarker disease activity and autoantibody status in patients with rheumatoid arthritis on tapering DMARD treatment. Ann. Rheum. Dis. 75 (2016), pp. 1637–1644.

[51] A. Paradowska-Gorycka, B. Raszkiewicz, M. Jurkowska, A. Felis-Giemza, K. Romanowska-Prochnicka, M. Manczak et al. Association of single nucleotide polymorphisms in the IL27 gene with rheumatoid arthritis. Scand. J. Immunol. 80 (2014), pp. 298–305.

[52] H. Shen, L. Xia, W. Xiao, J. Lu. Increased levels of interleukin-27 in patients with rheumatoid arthritis. Arthritis Rheum. 63 (2011), pp. 860–861.

[53] X. Lai, H. Wang, J. Cao, Y. Li, Y. Dai, Y. Xiang et al. Circulating IL-27 is elevated in rheumatoid arthritis patients. Molecules, 21 (2016)

[54] S. Tanida, H. Yoshitomi, M. Ishikawa, T. Kasahara, K. Murata, H. Shibuya et al. IL-27-producing CD14(+) cells infiltrate inflamed joints of rheumatoid arthritis and regulate inflammation and chemotactic migration. Cytokine, 55 (2011), pp. 237–244.

[55] C.K. Wong, D.P. Chen, L.S. Tam, E.K. Li, Y.B. Yin, C.W.K. Lam. Effects of inflammatory cytokine IL-27 on the activation of fibroblast-like synoviocytes in rheumatoid arthritis. Arthritis Res. Ther. 12 (2010). R129-R.

[56] W. Niedbala, B. Cai, X. Wei, A. Patakas, B.P. Leung, I.B. McInnes et al. Interleukin 27 attenuates collagen-induced arthritis. Ann. Rheum. Dis. 67 (2008), pp. 1474–1479.

[57] G.W. Jones, M. Bombardieri, C.J. Greenhill, L. McLeod, A. Nerviani, V. Rocher-Ros et al. Interleukin-27 inhibits ectopic lymphoid-like structure development in early inflammatory arthritis. J. Exp. Med. 212 (2015), pp. 1793–1802.

[58] D.Y. Wang, R. Fulthorpe, S.N. Liss, E.A. Edwards. Identification of estrogen-responsive genes by complementary deoxyribonucleic acid microarray and characterization of a novel early estrogen-induced gene: EEIG1. Mol. Endocrinol. 18 (2004), pp. 402–411.

[59] H.K. Choi, H.R. Kang, E. Jung, T.E. Kim, J.J. Lin, S.Y. Lee. Early estrogen-induced gene 1, a novel RANK signaling component, is essential for osteoclastogenesis. Cell Res. 23 (2013), pp. 524–536.

[60] D. Zhang, L. Aravind. Identification of novel families and classification of the C2 domain superfamily elucidate the origin and evolution of membrane targeting activities in eukaryotes. Gene, 469 (2010), pp. 18–30.

[61] P. Seite, K. Huebner, M.F. Rousseau-Merck, R. Berger, H.J. Thiesen. Two human genes encoding zinc finger proteins, ZNF 12 (KOX 3) and ZNF 26 (KOX 20), map to chromosome 7p22-p21 and 12q24.33, respectively. Hum. Genet. 86 (1991), pp. 585–590.

[62] Y. Li, M. Oosting, S.P. Smeekens, M. Jaeger, R. Aguirre-Gamboa, K.T.T. Le et al. A functional Genomics approach to understand variation in cytokine production in humans. Cell, 167 (2016). 1099-110.e14.

[63] D.R. Zerbino, P. Achuthan, W. Akanni, M.R. Amode, D. Barrell, J. Bhai et al. Ensembl 2018. Nucleic Acids Res. 46 (2018), pp. D754-D761.

[64] X.M. Teitsma, J.W.G. Jacobs, M. Mokry, M.E.A. Borm, A. Petho-Schramm, J.M. van Laar et al. Identification of differential co-expressed gene networks in early rheumatoid arthritis achieving sustained drug-free remission after treatment with a tocilizumab-based or methotrexate-based strategy. Arthritis Res. Ther. 19 (2017), p. 170.

[65] K.F. Baker, A. Skelton, D. Lendrem, B. Thompson, A.G. Pratt, J.D. Isaacs. OP0043 Predictors of drug-free remission in rheumatoid arthritis: Results from the prospective biomarkers of remission in rheumatoid arthritis (BIORRA) study [abstract]. Ann. Rheum. Dis. 77 (2018), p. 73.

