# Supplementary material for: Predicting drug-free remission in rheumatoid arthritis: A prospective interventional cohort study
Source: J Autoimmun. 2019 Dec;105:102298. doi: 10.1016/j.jaut.2019.06.009 (PMC6891251; doi:10.1016/j.jaut.2019.06.009)
Supplement: Multimedia component 1 [file mmc1.docx]

**Predicting drug-free remission in rheumatoid arthritis: a prospective interventional cohort study**

**SUPPLEMENTARY MATERIAL**

**Contents**

1. Supplementary Figure
2. Supplementary Tables
3. **SUPPLEMENTARY FIGURE**

**
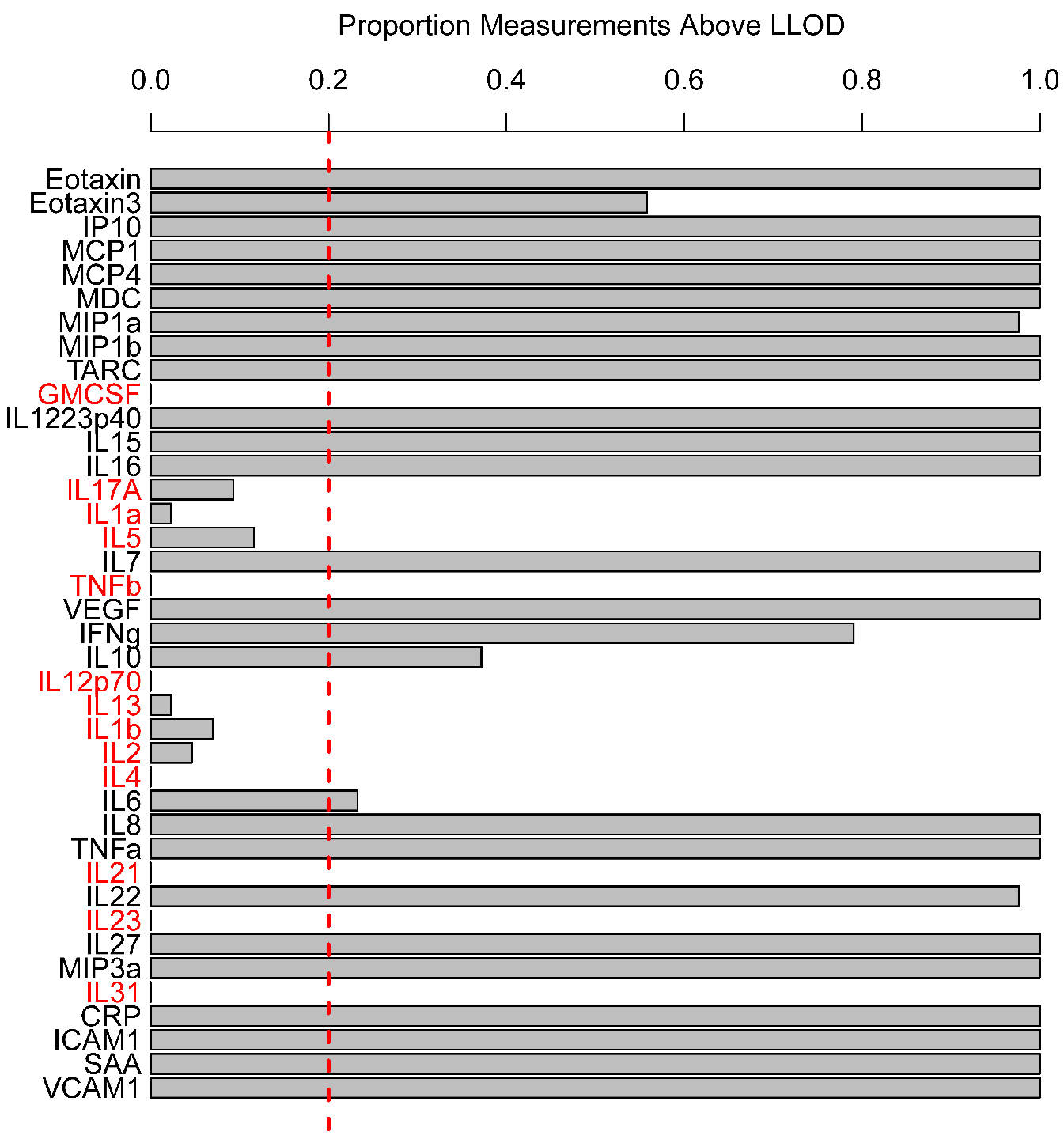
**

**Supplementary Figure S1**. **Circulating cytokines and chemokines measured by electrochemiluminescence in serum samples.** Analytes where <20% of measurements fell above the lower limit of detection (LLOD - highlighted in red) were excluded from the analysis. CCL: C-C motif chemokine ligand; CXCL: C-X-C motif chemokine ligand; GM-CSF: granulocyte-macrophage colony-stimulating factor; hsCRP: high-sensitivity C-reactive protein; ICAM: intercellular adhesion molecule; IFN: interferon; IL: interleukin; MCP: monocyte chemoattractant protein; MDC: macrophage-derived chemokine; MIP: macrophage inhibitory protein; IP-10: interferon-γ induced protein 10kDa; SAA: serum amyloid A; TARC: thymus and activation-regulated chemokine; TNF: tumour necrosis factor; VCAM: vascular cell adhesion molecule; VEGF: vascular endothelial growth factor.

1. **SUPPLEMENTARY TABLES**

**Supplementary Table S1**. **Pre-specified clinical variables recorded at baseline visit.** ACR: American College of Rheumatology; CRP: C-reactive protein; DAS28: disease activity score in 28 joints; ESR: erythrocyte sedimentation rate; EULAR: European league Against Rheumatism; HAQ-DI: Health Assessment Questionnaire Disability Index.

| Baseline variable | Data type |
| --- | --- |
| Age | Continuous |
| Sex | Binary |
| Tender (28) joint count | Discrete |
| Swollen (28) joint count | Discrete |
| Patient arthritis visual analogue score (range 0-100) | Continuous |
| ESR | Continuous |
| CRP | Continuous |
| Rheumatoid factor positive | Binary |
| Anti-citrullinated peptide antibody positive | Binary |
| DAS28-CRP | Continuous |
| DAS28-ESR | Continuous |
| Fulfilment of ACR/EULAR Boolean remission | Binary |
| HAQ-DI (range 0-3) | Continuous |
| Patient global health score (range 0-100) | Continuous |
| Patient pain score (range 0-100) | Continuous |
| Year of rheumatoid arthritis diagnosis | Continuous |
| Months from symptom onset to first rheumatology clinic review | Continuous |
| Months from first rheumatology clinic review to commencement of first DMARD | Continuous |
| Months since last change in DMARD therapy (dose and/or drug) | Continuous |
| Months since last glucocorticoid | Continuous |
| Smoking status | Categorical  (current/previous/never) |
| Weekly alcohol unit intake | Continuous |
| Methotrexate use | Categorical  (current/previous/never) |
| Sulfasalazine use | Categorical  (current/previous/never) |
| Hydroxychloroquine use | Categorical  (current/previous/never) |
| Other previous DMARDs | Free text |

**Supplementary Table S2. Adverse events recorded during the study.**

| Category | Adverse Event | Number of Events |
| --- | --- | --- |
| Infection | Nasopharyngitis | 12 |
|  | Pneumonia | 2 |
|  | Skin infection | 2 |
|  | Influenza-like illness | 1 |
|  | Herpes zoster | 1 |
|  | Oral herpes | 1 |
|  | Urinary tract infection | 1 |
| Respiratory | Breathlessness | 2 |
|  | Incidental finding of asbestos-related pleural plaque | 1 |
|  | Nasal polyposis | 1 |
| Metabolic | Hypercholesterolaemia | 3 |
|  | Increase in diabetes mellitus medications | 1 |
| Circulatory | Outpatient coronary imaging | 2 |
|  | Increase in ischaemic heart disease medications | 1 |
| Musculoskeletal | Arthritis flare | 24 |
|  | Elbow epicondylitis | 2 |
|  | Muscle cramp | 1 |
|  | Lower back pain | 1 |
|  | Myalgia & lethargy following intravenous bisphosphonate | 1 |
|  | Pain around knee replacement | 1 |
|  | Sialadenitis | 1 |
| Skin | Actinic keratosis | 3 |
|  | Basal cell carcinoma | 2 |
|  | Dry skin | 1 |
|  | Itch | 1 |
| Gastrointestinal | Diarrhoea | 2 |
|  | Abdominal pain | 2 |
|  | Irritable bowel syndrome | 1 |
|  | Inguinal hernia | 1 |
|  | Routine elective screening colonoscopy | 1 |
|  | Fatty liver change on ultrasound | 1 |
| Ophthalmological | Red/dry eyes | 2 |
|  | Elective phacoemulsification | 2 |
|  | Elective ocular punctoplasty | 1 |
| Other | Influenza vaccination | 10 |
|  | Fall | 5 |
|  | Pneumococcal vaccination | 1 |
|  | Dental extraction | 1 |
|  | Fatigue | 1 |
|  | Hay fever | 1 |

**Supplementary Table S3**. **Association of baseline clinical variables with occurrence of arthritis flare following disease-modifying anti-rheumatic drug (DMARD) cessation by univariate Cox regression.** For continuous variables, hazard ratios (HR) and Cox regression coefficients (B) are presented for a 1-unit change in that variable. Statistical significance calculated by the Wald test. ACPA: anti-citrullinated peptide antibody; ACR: American College of Rheumatology; CI: confidence interval; CRP: C-reactive protein; DAS28: disease activity score in 28 joints; ESR: erythrocyte sedimentation rate; HAQ-DI: Health Assessment Questionnaire Disability Index; RhF: rheumatoid factor; VAS: visual analogue scale.

| Variable | B | HR_flare_ | 95% CI | Univariate p |
| --- | --- | --- | --- | --- |
| Symptom duration prior to first rheumatology review (months) | 0.036 | 1.04 | 1.00 – 1.07 | 0.032 |
| Current number of DMARDs | 0.704 | 2.02 | 1.06 – 3.86 | 0.033 |
| Double seropositive | 0.836 | 2.31 | 1.01 – 5.25 | 0.046 |
| Cumulative number of DMARDs since diagnosis | 0.401 | 1.49 | 0.98 – 2.27 | 0.060 |
| Months since last change in DMARD therapy | -0.015 | 0.98 | 0.97 – 1.00 | 0.067 |
| Male sex | 0.763 | 2.14 | 0.93 – 4.96 | 0.075 |
| Current hydroxychloroquine | 0.752 | 2.12 | 0.89 – 5.04 | 0.089 |
| Baseline ACR/EULAR Boolean remission | -0.692 | 0.50 | 0.22 – 1.14 | 0.098 |
| Baseline VAS_patient_ | 0.032 | 1.03 | 0.99 – 1.07 | 0.100 |
| RhF positive | 0.698 | 2.01 | 0.85 – 4.77 | 0.113 |
| Months from first rheumatology review to starting first DMARD | 0.007 | 1.01 | 1.00 – 1.02 | 0.141 |
| ACPA positive | 0.630 | 1.88 | 0.79 – 4.44 | 0.152 |
| Current methotrexate | 1.422 | 4.14 | 0.56 – 30.84 | 0.165 |
| Weekly alcohol unit intake | 0.033 | 1.03 | 0.99 – 1.08 | 0.167 |
| Disease duration (years) | 0.034 | 1.03 | 0.99 – 1.09 | 0.172 |
| Baseline tender (28) joint count | -0.700 | 0.50 | 0.16 – 1.53 | 0.222 |
| Either RhF or ACPA positive | 0.612 | 1.84 | 0.68 – 4.99 | 0.229 |
| Ever smoker | -0.405 | 0.67 | 0.29 – 1.52 | 0.334 |
| Months since last steroid | -0.010 | 0.99 | 0.97 – 1.01 | 0.354 |
| Baseline DAS28-ESR remission | 0.579 | 1.79 | 0.42 – 7.67 | 0.436 |
| Baseline CRP (mg/L) | 0.055 | 1.06 | 0.92 – 1.21 | 0.440 |
| Baseline HAQ-DI | -0.295 | 0.74 | 0.32 – 1.74 | 0.495 |
| Baseline ESR (mm/hr) | 0.009 | 1.01 | 0.98 – 1.04 | 0.526 |
| Age (years) | 0.011 | 1.01 | 0.97 – 1.05 | 0.569 |
| Current smoker | -0.314 | 0.73 | 0.17 – 3.12 | 0.671 |
| Current sulfasalazine | -0.109 | 0.90 | 0.35 – 2.28 | 0.819 |
| Baseline DAS28-CRP | 0.080 | 1.08 | 0.45 – 2.60 | 0.858 |
| Baseline swollen (28) joint count | 0.049 | 1.05 | 0.54 – 2.02 | 0.885 |
| Baseline DAS28-ESR | 0.019 | 1.02 | 0.63 – 1.64 | 0.938 |

**Supplementary Table S4**. **Association of clinical variables with occurrence of arthritis flare following disease-modifying anti-rheumatic drug (DMARD) cessation by univariate Cox regression.** Statistical significance calculated by the Wald test.

| Variable | B | HR_flare_ | 95% CI | p |
| --- | --- | --- | --- | --- |
| Total synovial greyscale score | 0.13 | 1.14 | 0.93 – 1.40 | 0.22 |
| Total tenosynovial greyscale score | 0.01 | 1.01 | 0.52 – 1.97 | 0.98 |
| Total erosion score | 0.16 | 1.17 | 0.96 – 1.43 | 0.11 |
| Number of joints with greyscale score ≥ 1 | 0.09 | 1.10 | 0.77 – 1.56 | 0.61 |
| Number of joints with greyscale score ≥ 2 | 0.40 | 1.49 | 0.91 – 2.43 | 0.11 |
| Number of joints with erosions | 0.12 | 1.13 | 0.82 – 1.56 | 0.47 |

**Supplementary Table S5**. **Association of the circulating concentration of serum protein biomarkers at baseline with occurrence of arthritis flare following disease-modifying anti-rheumatic drug (DMARD) cessation by univariate Cox regression.** Hazard ratios (HR) and Cox regression coefficients (B) are presented for a 1-unit change in log-transformed biomarker concentration. Statistical significance calculated by the Wald test. CI: confidence interval; hsCRP: high-sensitivity C-reactive protein; ICAM: intercellular adhesion molecule; IFN: interferon; IL: interleukin; MCP: monocyte chemoattractant protein; MDC: macrophage-derived chemokine; MIP: macrophage inhibitory protein; IP-10: interferon-γ induced protein 10kDa; SAA: serum amyloid A; TARC: thymus and activation-regulated chemokine; TNF: tumour necrosis factor; VCAM: vascular cell adhesion molecule; VEGF: vascular endothelial growth factor.

| Variable | B | HR_flare_ | 95% CI | Univariate p |
| --- | --- | --- | --- | --- |
| ln(MCP1+1) | 2.212 | 9.13 | 1.97 – 42.32 | 0.005 |
| ln(hsCRP+1) | 0.426 | 1.53 | 1.02 – 2.31 | 0.042 |
| ln(Eotaxin+1) | 1.386 | 4.00 | 0.97 – 16.5 | 0.055 |
| ln(IL6+1) | 0.730 | 2.08 | 0.97 – 4.45 | 0.060 |
| ln(TNFa+1) | 1.273 | 3.57 | 0.93 – 13.7 | 0.063 |
| ln(IP10+1) | 0.592 | 1.81 | 0.97 – 3.38 | 0.064 |
| ln(IL10+1) | 1.737 | 5.68 | 0.65 – 49.8 | 0.117 |
| ln(IL27+1) | 0.948 | 2.58 | 0.78 – 8.53 | 0.120 |
| ln(MCP4+1) | 0.522 | 1.69 | 0.84 – 3.38 | 0.140 |
| ln(IL15+1) | 1.572 | 4.82 | 0.56 – 41.6 | 0.153 |
| ln(Eotaxin3+1) | 0.310 | 1.36 | 0.81 – 2.28 | 0.238 |
| ln(VCAM1+1) | 0.654 | 1.92 | 0.55 – 6.72 | 0.306 |
| ln(IL16+1) | 0.694 | 2.00 | 0.52 – 7.65 | 0.311 |
| ln(IL7+1) | 0.628 | 1.87 | 0.51 – 6.84 | 0.342 |
| ln(IL22+1) | -0.502 | 0.61 | 0.20 – 1.87 | 0.384 |
| ln(MIP1a+1) | -0.485 | 0.62 | 0.19 – 2.04 | 0.427 |
| ln(TARC+1) | 0.283 | 1.33 | 0.64 – 2.76 | 0.448 |
| ln(MIP1b+1) | 0.342 | 1.41 | 0.47 – 4.17 | 0.538 |
| ln(MDC+1) | -0.376 | 0.69 | 0.15 – 3.07 | 0.623 |
| ln(IL8+1) | 0.171 | 1.19 | 0.55 – 2.57 | 0.664 |
| ln(IFNg+1) | 0.074 | 1.08 | 0.73 – 1.59 | 0.712 |
| ln(VEGF+1) | -0.043 | 0.96 | 0.46 – 1.99 | 0.909 |
| ln(MIP3a+1) | 0.033 | 1.03 | 0.58 – 1.85 | 0.912 |
| ln(SAA+1) | 0.017 | 1.02 | 0.66 – 1.56 | 0.940 |
| ln(ICAM1+1) | 0.033 | 1.03 | 0.34 – 3.15 | 0.954 |
| ln(IL1223p40+1) | 0.019 | 1.02 | 0.47 – 2.21 | 0.961 |

**Supplementary Table S6**. **Association between baseline gene expression and time-to-flare following disease-modifying anti-rheumatic drug (DMARD) cessation by univariate Cox regression**. An unadjusted significance threshold of p<0.001 was calculated using the likelihood ratio test. Hazard ratios (HR) and Cox regression coefficients (B) are presented for a 1-unit change in normalised log-transformed gene expression value. CI: confidence interval; HGNC: HUGO gene nomenclature committee. LincRNA: long intergenic non-coding RNA.

| Ensembl gene ID | B | HR_flare_ | 95% CI | Unadjusted p | HGNC symbol | Description |
| --- | --- | --- | --- | --- | --- | --- |
| ENSG00000102362 | 1.46 | 4.30 | 1.93 – 9.58 | 2.04E-05 | *SYTL4* | synaptotagmin like 4 |
| ENSG00000247033 | 0.61 | 1.84 | 1.33 – 2.54 | 4.11E-05 |  | (novel antisense) |
| ENSG00000276571 | -0.91 | 0.40 | 0.25 – 0.65 | 7.33E-05 |  | (novel antisense) |
| ENSG00000204965 | -0.78 | 0.46 | 0.31 – 0.68 | 9.04E-05 | *PCDHA5* | protocadherin alpha 5 |
| ENSG00000241146 | -0.89 | 0.41 | 0.27 – 0.63 | 1.44E-04 | *RPL7P41* | ribosomal protein L7 pseudogene 41 |
| ENSG00000250030 | 0.97 | 2.63 | 1.58 – 4.36 | 2.15E-04 |  | (novel processed pseudogene) |
| ENSG00000213296 | -1.01 | 0.37 | 0.20 – 0.66 | 2.50E-04 |  | (known processed pseudogene) |
| ENSG00000229619 | 1.67 | 5.30 | 2.05 – 13.7 | 2.72E-04 | *MBNL1-AS1* | muscleblind-like protein 1 - antisense RNA 1 |
| ENSG00000125046 | -0.99 | 0.37 | 0.22 – 0.61 | 2.97E-04 | *SSUH2* | suppressor of stomatin mutant uncoordination (ssu-2) homolog (C. elegans) |
| ENSG00000182489 | -0.63 | 0.53 | 0.38 – 0.75 | 3.17E-04 | *XKRX* | Kell Blood Group Complex Subunit-Related, X-Linked |
| ENSG00000144366 | 0.63 | 1.87 | 1.30 – 2.70 | 4.84E-04 | *GULP1* | engulfment adaptor PTB domain containing 1 |
| ENSG00000237473 | 1.23 | 3.42 | 1.58 – 7.39 | 5.28E-04 |  | (known lincRNA) |
| ENSG00000228010 | -1.38 | 0.25 | 0.11 – 0.56 | 6.02E-04 |  | (novel antisense) |
| ENSG00000250827 | -0.76 | 0.47 | 0.30 – 0.74 | 7.86E-04 | *MFSD4BP1* | major facilitator superfamily domain containing 4B pseudogene 1 |
| ENSG00000042286 | 2.32 | 10.2 | 2.42 – 42.6 | 7.94E-04 | *AIFM2* | apoptosis inducing factor, mitochondria associated 2 |
| ENSG00000231305 | -1.41 | 0.24 | 0.11 – 0.56 | 7.99E-04 |  | (known antisense) |
| ENSG00000255330 | 0.98 | 2.66 | 1.32 – 5.36 | 8.33E-04 | *SOGA3* | suppressor of glucose, autophagy associated (SOGA) family member 3 |
| ENSG00000227070 | 0.77 | 2.15 | 1.41 – 3.28 | 8.47E-04 |  | (novel antisense) |
| ENSG00000162636 | 2.56 | 12.9 | 2.57 – 64.5 | 9.14E-04 | *FAM102B* | family with sequence similarity 102 member B |

**Supplementary Table S7**. **Association of baseline gene expression and time-to-flare following disease-modifying anti-rheumatic drug (DMARD) cessation in a final 11-variable backward stepwise multivariate Cox regression model**. Hazard ratios (HR) and Cox regression coefficients (B) are presented for a 1-unit change in normalised log-transformed gene expression value. Statistical significance calculated by the Wald test. CI: confidence interval; HGNC: HUGO gene nomenclature committee.

| Ensembl gene ID | B | HR_flare_ | 95% CI | Multivariate p | HGNC symbol | Description |
| --- | --- | --- | --- | --- | --- | --- |
| ENSG00000228010 | -4.15 | 0.02 | 0.00 – 0.14 | 2.24E-04 |  | (novel antisense) |
| ENSG00000162636 | 6.97 | 1060 | 22.6 – 50000 | 3.88E-04 | *FAM102B* | family with sequence similarity 102 member B |
| ENSG00000227070 | 1.78 | 5.94 | 2.08 – 16.9 | 8.63E-04 |  | (novel antisense) |
| ENSG00000204965 | -1.69 | 0.18 | 0.07 – 0.52 | 1.45E-03 | *PCDHA5* | protocadherin alpha 5 |
| ENSG00000229619 | 2.93 | 18.7 | 2.90 – 121 | 2.08E-03 | *MBNL1-AS1* | muscleblind-like protein 1 - antisense RNA 1 |
| ENSG00000247033 | 1.18 | 3.26 | 1.53 – 6.98 | 2.29E-03 |  | (novel antisense) |
| ENSG00000125046 | -1.49 | 0.23 | 0.09 – 0.60 | 2.62E-03 | *SSUH2* | suppressor of stomatin mutant uncoordination (ssu-2) homolog (C. elegans) |
| ENSG00000241146 | -1.32 | 0.27 | 0.10 – 0.71 | 7.96E-03 | *RPL7P41* | ribosomal protein L7 pseudogene 41 |
| ENSG00000276571 | -0.83 | 0.44 | 0.18 – 1.07 | 7.07E-02 |  | (novel antisense) |
| ENSG00000250827 | 0.71 | 2.04 | 0.89 – 4.69 | 9.42E-02 | *MFSD4BP1* | major facilitator superfamily domain containing 4B pseudogene 1 |
| ENSG00000042286 | 1.75 | 5.78 | 0.68 – 49.3 | 1.09E-01 | *AIFM2* | apoptosis inducing factor, mitochondria associated 2 |

**Supplementary Table S8**. **Sensitivity analysis of baseline variables across all domains with time-to-flare following DMARD-cessation in a backward stepwise multivariate Cox regression model, following categorisation of the single ankle/foot synovitis patient as remission**. For continuous variables, hazard ratios (HR) and the Cox regression coefficients (B) are presented for a 1-unit change in that variable. Statistical significance calculated by the Wald test. ACR: American College of Rheumatology; CI: confidence interval; EULAR: European League Against Rheumatism; FAM102B: family with sequence similarity 102 member B IL: interleukin; RhF: rheumatoid factor.

| Variable | B | HR_flare_ | 95% CI | p |
| --- | --- | --- | --- | --- |
| *ENSG00000228010* | -1.826 | 0.16 | 0.06 – 0.43 | <0.001 |
| *ENSG00000227070* | 1.091 | 2.98 | 1.74 – 5.10 | <0.001 |
| ACR/EULAR Boolean remission | -1.506 | 0.22 | 0.08 – 0.59 | 0.002 |
| ln(IL27+1) | 2.002 | 7.40 | 1.80 – 30.41 | 0.005 |
| *FAM102B* | 2.977 | 19.62 | 2.35 – 163.59 | 0.006 |
| RhF positive | 0.776 | 2.17 | 0.79 – 6.02 | 0.135 |

**Supplementary Table S9**. **Sensitivity analysis of composite biomarker score performance following categorisation of the single ankle/foot synovitis patient as remission**. Identical variable coefficients and threshold were used as per the main analysis. CI: confidence interval; ROC_AUC_: area under the receiver operating characteristic curve.

| Parameter | Value | 95% CI |
| --- | --- | --- |
| Sensitivity | 0.91 | 0.77 – 1.00 |
| Specificity | 0.90 | 0.75 – 1.00 |
| Positive predictive value | 0.91 | 0.80 – 1.00 |
| Negative predictive value | 0.90 | 0.78 – 1.00 |
| ROC_AUC_ | 0.94 | 0.88 – 1.00 |
